# Predicting immunotherapy efficacy in endometrial cancer: focus on the tumor microenvironment

**DOI:** 10.3389/fimmu.2024.1523518

**Published:** 2025-01-20

**Authors:** Liubov A. Tashireva, Irina V. Larionova, Nikita A. Ermak, Anastasia A. Maltseva, Ekaterina I. Livanos, Anna Yu. Kalinchuk, Marina N. Stakheyeva, Larisa A. Kolomiets

**Affiliations:** ^1^ Laboratory of Molecular Therapy of Cancer, Cancer Research Institute, Tomsk National Research Medical Center, Russian Academy of Sciences, Tomsk, Russia; ^2^ Laboratory of Translational Cellular and Molecular Biomedicine, National Research Tomsk State University, Tomsk, Russia; ^3^ Laboratory of Molecular Oncology and Immunology, Cancer Research Institute, Tomsk National Research Medical Center, Russian Academy of Sciences, Tomsk, Russia; ^4^ Department of Gynecology, Cancer Research Institute, Tomsk National Research Medical Center, Russian Academy of Sciences, Tomsk, Russia

**Keywords:** endometrial cancer, immunotherapy, immune checkpoint inhibitors, tumor microenvironment, predictive biomarkers

## Abstract

Immunotherapy represents a groundbreaking therapeutic approach, based on the immune system’s intrinsic capacity to interfere with tumor progression, that opens the horizons in the treatment of endometrial cancer. However, the clinical efficacy of immunotherapy is hampered by the development of resistance in patients. The resistance to immunotherapy is multifactorial mechanism, encompassed genetic and epigenetic alterations in tumor cells modulating immune checkpoint molecules, resulted in escaping immune surveillance. The tumor microenvironment can orchestrate an immunosuppressive milieu, attenuating the immune response and facilitating tumor progression. To overcome immunotherapeutic resistance in endometrial cancer we must bring to light the mechanisms of intricate interplay between neoplastic cells, the host immune system, and the tumor microenvironment. The identification of predictive biomarkers for immunotherapeutic response and the innovative agents capable of reversing resistance pathways must be developed. Our review summarizes accumulated data on the role of cells of the tumor microenvironment and their regulatory molecules in the mechanisms underlying therapeutic effects of immune checkpoint inhibitors, including resistance to therapy. Major question we raise here – which group of patients is the most favorable to achieve durable immunotherapy response in endometrial cancer?

## 1 Introduction

Endometrial cancer (EC) represents a significant public health challenge globally. Based on the World Cancer Research Fund International data (2020), EC ranks sixth most prevalent cancer in women and the fifteenth in the whole population. Approximately 417,000 new EC cases and 97,370 related fatalities are revealed annually worldwide (1). There is an observed upward trend in both the incidence and mortality rates associated with EC. By 2044, the incidence of EC is projected to be exceeded 600,000 cases annually, that will account around 48% increase starting from 2019 (2, 3).

Therapeutic interventions in the early-stage EC can be markedly efficacious. The five-year survival rate for patients diagnosed at stage I is approximately 96%, at stage II – between 80-90%. However, the survival rate significantly declined to 20-25% in metastatic EC (4). The conventional regimen for the treatment of advanced stage EC includes a combination therapy with paclitaxel and carboplatin, which allowed to achieve a median overall survival (OS) equal to 37 months and an objective response rate of 52%. Disease progression is observed in nearly half of the patients with advanced EC within the first-year post-treatment. At the same time, second-line chemotherapeutic treatment does not demonstrate substantial efficacy (5). In light of these challenges, immunotherapy – specifically, immune checkpoint inhibitors (ICIs) – emerges as a promising approach to amplify the effectiveness of the conventional treatment in endometrial cancer (6, 7) (**Table 1**). PD-1/PD-L1 axis plays essential role in immune escape mechanisms and is reported the most effective target for ICI therapy (8). Herewith, the expression and activity of PD-L1 in tumor is under rigorous control accompanied by the complex molecular mechanisms including genomic amplification, epigenetic regulation, transcriptional regulation, translational regulation and posttranslational modification (9, 10).

**Table 1:** Efficacy of immune checkpoint therapy in endometrial cancer.

Agents	Target	Type of EC	Efficacy	Trial number
Pembrolizumab	PD-1	MSI, dMMR, recurrent or metastatic EC	Complete response - 20%; Partial response - 53%	NCT01876511
Nivolumab	PD-1	MSI, dMMR, recurrent EC	Complete response - 15%; Partial response - 23%; Reduction in tumor size - 53%	NCT02465060
Durvalumab	PD-L1	MSI, dMMR, advanced EC	Objective Response Rate - 47%; Median of progression free survival - 8.3 months	NCT02725489
Atezolizumab	PD-L1	MSI, recurrent or advanced EC	Partial response - 13%; Median of Overall survival - 9.6 months; Median of progression free survival - 1.7 months	NCT01375842
Avelumab	PD-L1	MSI, recurrent or advanced EC	Partial response - 26.7%; Progression free survival after 6 months - 40%	NCT02912572
Tremelimumab + durvalumab	CTLA-4; PD-L1	Recurrent EC	Objective Response Rate - 40%	NCT03015129
INCAGN02385	LAG-3	MSI, recurrent or metastatic EC	Unpublished data	NCT03538028

EC, endometrial cancer; PD-1, programmed cell death 1; PD-L1, programmed cell death ligand 1; MSI, microsatellite instability; dMMR, deficient DNA mismatch repair; CTLA-4, cytotoxic T-lymphocyte-associated protein 4; LAG-3, lymphocyte-activation gene 3.

The effectiveness of immunotherapy in monotherapy and in combination varies from 26.7% to 63.6%, respectively. Data on clinical trials involved in studying ICI effects were extensively collected in systemic review (11). Predicting tumor response to ICIs is possible due to an effective differentiation between so-named “cold” (immune-desert) and “hot” (immune-inflamed) tumor phenotypes. Tumor intrinsic factors and tumor immune microenvironment characteristics underlie the concept of “cold” and “hot” tumors. Key factors related to the durable response rate and efficiency of ICIs in EC include high levels of T cell infiltration, increased PD-L1 expression, and high tumor mutation burden (TMB) or MSI-H/dMMR status of tumors (12).

Despite the progress made in improving the immunotherapy efficacy for patients with advanced or recurrent EC, there is still a considerable proportion of patients who do not respond to the treatment. It is necessary to search for and implement highly accurate biomarkers for immunotherapy application in patients with EC. In the present review, we collected and described data accumulated for several factors, which allow predicting the response to immunotherapy in EC patients: genetic alterations, transcriptomic signatures of immune cells, transcriptomic immune-related signatures and proteomic signatures.

## 2 Genetic alterations

The integration of molecular genetics into clinical oncology has revolutionized the classification of molecular subtypes in tumors, including EC. Pioneering work made by Levine et al. resulted in the stratification of EC into four genetic subtypes with distinct prognostic implications (13). These subtypes are defined as follows: POLE mut (10%), with a high mutation rate in DNA polymerase epsilon; MSI-high (20%), indicating microsatellite instability; TP53 wt (52%) with wild-type p53 status; and TP53 mut (18%), with TP53 gene mutations. POLE mut shows elevated T effector and interferon (IFN) signatures, suggesting a strong immune response and reduced innate resistance to anti-PD-1 therapy, making it potentially more responsive to immunotherapy. In contrast, TP53 wt has lower T effector and IFN-related gene expression, possibly indicating resistance to ICIs. This highlights the importance of molecular profiling not only for prognosis, but also in predicting immunotherapy responses (14).

Expanding this genetic framework, molecular subtypes of EC based on immune suppression gene signatures were determined in recent study (15). These signatures overlap pathways involved in T cell exhaustion, Treg function, myeloid-derived suppressor cell (MDSC) activity, transforming growth factor-beta (TGF-β) signaling, and IFN-γ signaling. This analysis revealed four immune subtypes: the immune-activated subtype, characterized by strong expression of immune cell markers and immune checkpoint molecules, reflecting a robust anti-tumor immune environment; the immune-deficient subtype, defined by low immune gene expression and related to poorer clinical outcomes; the IFN-γ dominant subtype, marked by high expression of genes driving IFN-γ signaling, a key mediator of immune response; and the TGF-β dominant subtype, distinguished by elevated expression of genes of TGF-β signaling pathway, known for its role in immune modulation (15).

Several mutations found in tumor tissues of EC patients were demonstrated to be associated with response to immune checkpoint blockade (ICB) therapy. Thus, mutations in a *POLE* gene in patients with EC may result in the overexpression of PD-1 in tumor-infiltrating lymphocytes. This indicates that mutations in *POLE* can be predictive for the effectiveness of anti-PD-1 therapy in EC (16). Two cases of advanced ultra/hypermutated EC with mutations in *POLE* were described. These two patients who underwent radiotherapy plus chemotherapy followed by administration of nivolumab obtained a sustained clinical efficacy with a duration of seven months (17). The most common mutations indicative for EC patients were mutations in genes *PTEN*, *ARID1A*, and *PIK3CA* (18).

The correlation between mutations in *ARID1A*, a gene implicated in chromatin remodeling, and improved outcomes after ICB therapy is well-documented across various cancer types including EC (19). A comprehensive study across multiple cancer types demonstrated better survival rates in EC patients with *ARID1A* mutations who undergone ICB therapy (20). However, this study included only a small cohort (N=10) that was insufficient to establish a significant survival benefit. *ARID1A* mutations was associated with Treg infiltration and enhanced type-I IFN response pathway, and authors supposed that it could improve the immune response and survival rates in EC patients (20). *ARID1A* directly interacts with DNA mismatch repair protein MSH2, a key component of the DNA mismatch repair (MMR) system (21). The absence of *ARID1A* disrupts *MSH2* functionality, resulting in a high frequency of microsatellite instability (MSI-high). This process is often accompanied by an upregulation of PD-L1, and tumors harboring *ARID1A* mutations typically respond to the treatment with ICIs better than tumors without mutations in *ARID1A* (22, 23). However, this correlation does not produce a desired effect in microsatellite stable (MSS) endometrial carcinoma, where *ARID1A* deficiency does not lead to notable PD-L1 expression or anti-tumor immune infiltration (24). Authors of recent study concluded that the predictive value of *ARID1A* mutations for ICI therapy response can be limited in EC due to the insufficiency of *ARID1A*-negative tumors with MSI-high status (24). This is out of sync with studies on other cancer types that defined mutation in *ARID1A* as a predictive biomarker for ICI response.

A mutational signature of a DNA polymerase delta catalytic subunit gene (*POLD1*), specifically the p.D402N variant, was identified in the tumor tissue of patient with EC (25). A comprehensive whole-exome and transcriptomic analysis of tumor tissues revealed a pronounced hypermutated state of tumors along with a T cell-inflamed gene expression profile in patients with EC. These features collectively served as prognostic indicator for the efficacy of pembrolizumab therapy (25). In a single-arm Phase 2 clinical trial of nivolumab, patients with dMMR (deficient DNA mismatch repair) uterine or ovarian cancers who had *MEGF8* or *SETD1B* somatic mutations experienced better 24-week PFS (26).

These findings highlight the complex interplay between genetic alterations and the immune landscape in EC. The molecular deciphering of heterogeneous EC subtypes do not only help in prognostication but also paves the way for personalized immunotherapeutic approaches, which will potentially improve patient outcomes.

## 3 Transcriptomic signatures of immune cells

Among various predictors of immunotherapy response in EC, gene signatures related to immune cell types are the most prevalent. In analysis of 539 EC specimens, a prognostic model was built based on four ICI-associated genes: *LINC01871*, *CXCL13*, *IGKJ5*, and *LINC01281* (27). Tumor tissues from patients with a lower risk score had an abundant infiltration with immune effector cells and were classified as having an immune-inflamed phenotype. These patients also showed high immunophenoscore (specifically, high CTLA4 and PD1 scores), indicating a likely better response to immunotherapy (27). Another study found that EC patients with a high novel immune risk score (NIRS), included *CTSW*, *CD3D*, and *CD48* genes, exhibited diminished levels of immune cell infiltration, fewer gene mutations, and lower expression of various immune checkpoints (28). Authors suggested that high NIRS that are related to diminished CD8+ T cell infiltration can be indicative for a decreased responsiveness to immunotherapy (28).

A subset of tumor-infiltrating CXCL8^hi^IL1B^hi^ macrophages was identified as a predictor for the durable response to immunotherapy in EC (29). A prognostic signature was developed using six genes from this macrophage subset – *SCL8A1*, *TXN*, *ANXA5*, *CST3*, *CD74*, and *NANS*. Patients with high-risk score had shorter survival and worse response to immunotherapy (29). The existence of dysfunctional (CD8+PD-1+) or terminally dysfunctional (CD8+PD-1+TOX+) T cells, along with their interaction with PD-L1+ cells, independently predicted a 24-month PFS in patients with dMMR uterine or ovarian cancers treated with nivolumab (26). In another study, CXCL9-secreting antigen-presenting and CXCL13-secreting follicular dendritic cells contributed to the formation of an immune-activated milieu in EC via the maintaining tertiary lymphoid structures (TLSs) (30). Complementary study indicated that the detection of TLSs within hematoxylin-eosin-stained sections correlated with improved PFS in patients with advanced dMMR endometrial cancer undergone second-line treatment with Durvalumab (31). To substantiate these preliminary findings and to delineate further the factors that can predict the response to Durvalumab, particularly in first-line settings, the analysis of larger patient cohorts can be suggested. Subsequent study revealed that the presence of cytotoxic T-cell response elements within the TME, including elevated IFN-γ and T-cell inflamed scores accompanied with high HLA class II expression, was related to an extended duration of response with pembrolizumab and lenvatinib in serous endometrial cancer (32). Another cells that may have clinical relevance in the context of anti-PD-1 therapy is T follicular helper (Tfh) (33). Classifying patients based on Tfh presence can predict the effectiveness of ICIs. Thus, high Tfh score was associated with favorable outcomes following anti-PD-1 therapy. A notably greater incidence of complete or partial response was observed in patients with abundant Tfh infiltration compared to those with minimal Tfh infiltration. Hereby, the stratification of patients according to Tfh levels can constitute an optimal way for enhancing the accuracy for administration of anti-PD-1 treatment in EC (33). In patients with MSI-H endometrial cancer treated with pembrolizumab, a significant increase in fibroblast and endothelial cell transcriptomic signatures was observed in non-responders compared to responders (34, 35). Specifically, the expression of *TAGLN* (fibroblast gene) and endothelial cell genes (*EMCN*, *KDR*, *MMRN1*, *MYCT1*, *PEAR1*, *PTPRB*, and *TEK*) was elevated as response decreased (34). Imaging mass cytometry analysis demonstrated similar trend of higher population of activated fibroblasts (SMA+, MFAP5+) and endothelial cells (CD31+) as well as regulatory T cells (CD4+FOXP3+) in non-responders (35).

Another study found that the effectiveness of immunotherapy is linked to both the presence and the functional state of certain immune cell markers (36).

**Figure 1:**
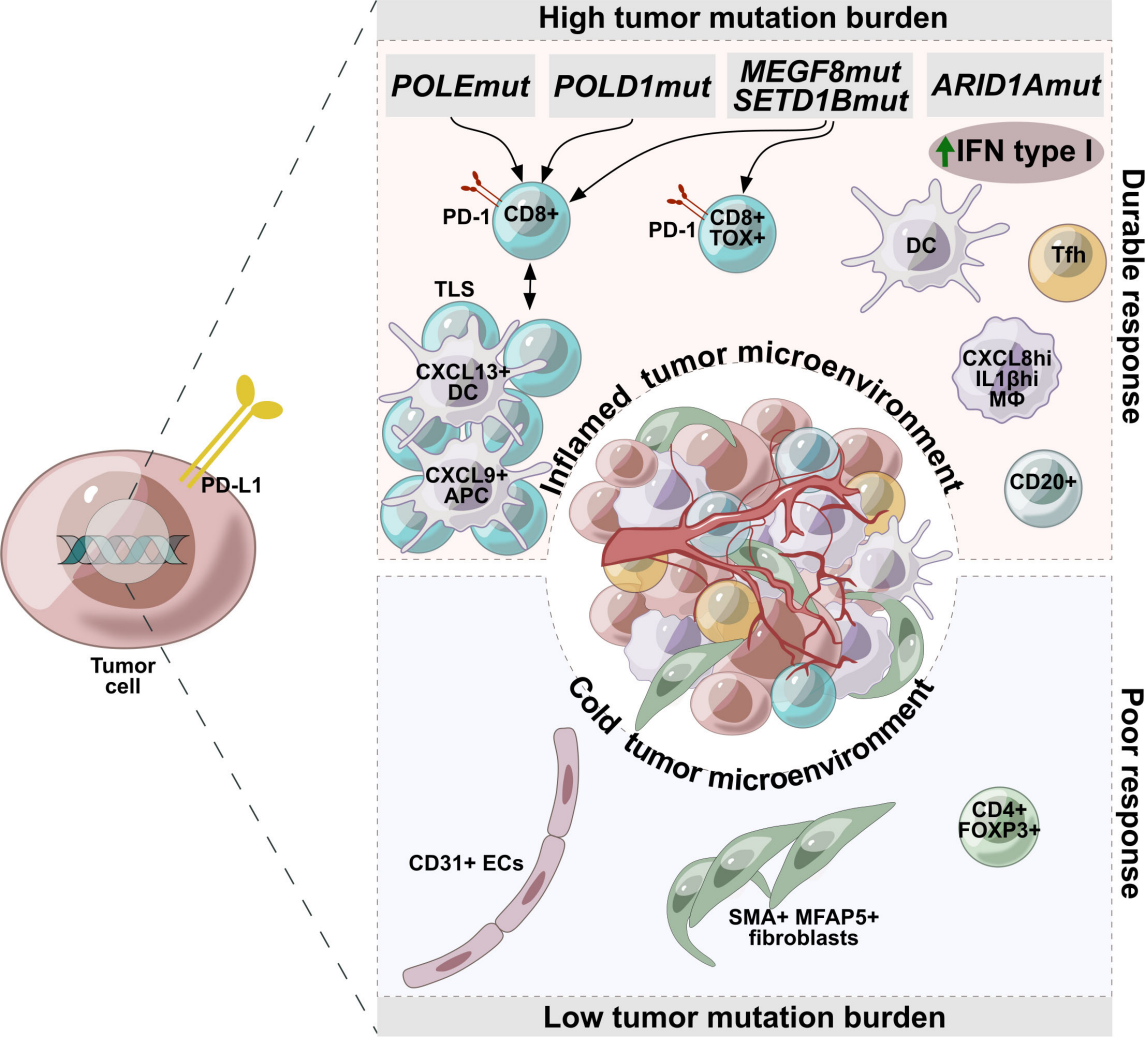
Factors associated with durable or poor response to immunotherapy in endometrial cancer. Tumors with high mutational burden are characterized by accumulation of Tfh, cytotoxic T-cell, dysfunctional (CD8+PD-1+) or terminally dysfunctional (CD8+PD-1+TOX+) T cells, CXCL8^hi^IL1B^hi^ macrophage subsets, and TLS presence, making these tumors potentially more sensitive to immunotherapy. Tumors with a low mutational burden are characterized by a cold microenvironment and are infiltrated with activated fibroblasts (SMA+, MFAP5+), endothelial cells (CD31+), regulatory T cells (CD4+FOXP3+), which are indicators of low efficacy of immunotherapy.

Thus, a balance in the composition and the activation state of the TME can be strongly associated with the response to immunotherapy in EC, and foremost in MSI-H tumors. A durable response to ICI were detected in patients with the TME strongly accumulated by Tfh, cytotoxic T-cell, dysfunctional (CD8+PD-1+) or terminally dysfunctional (CD8+PD-1+TOX+) T cells, CXCL8^hi^IL1B^hi^ macrophage subsets, and TLS presence. Tumor infiltration with activated fibroblasts (SMA+, MFAP5+) and endothelial cells (CD31+) as well as regulatory T cells (CD4+FOXP3+) was an indicator for marginal immunotherapy efficacy (**Figure 1**).

## 4 Transcriptomic immune-related signatures

Some gene signatures comprise genes which don’t directly belong to immune cell markers, but are associated with immune cell response. A prognostic model constructed using five genes associated with metabolic processes (*INPPP5K*, *PLPP2*, *MBOAT2*, *DDC*, and *ITPKA*) was related to immune cell infiltration and immune system functions in EC (37). It was correlated with the expression of immune checkpoints (CD279, CTLA4, and CD40) and predicted clinical outcomes of ICI therapy (37).

Another immune-related prognostic signature (IRPS) was designed based on the expression of two genes, *CCL13* and *KLRC1*. It was observed that patients exhibiting a lower IRPS demonstrated more favorable outcome from immunotherapy and more robust response to the treatment following various chemotherapeutic regimens (38). Authors utilized a panel of 34 immune cell dysfunction-related genes (ICDRGs) to calculate the Immune Phenotype Score (IPS), which helped to predict the clinical efficacy of ICIs in various EC patient cohorts (36). The findings indicated that patients with EC who had lower ICDRG score exhibited higher IPS, suggesting a greater sensitivity to immunotherapy compared to those with higher ICDRG score. In this study, the authors did not describe specific cell types associated with response to immunotherapy (36).

An immune signature, included following genes *CBLC*, *PLA2G2A*, *TNF*, *NR3C1*, *APOD*, *TNFRSF18*, and *LTB*, was an independent prognostic criterion for OS and outperformed conventional methods for staging of EC (39). Based on this gene signature, patients categorized at low-risk group had an increased sensitivity to ICI therapy (39). The majority of the genes composing this signature play essential roles in controlling the immune response. Specifically, c-Cbl (*CBLC* gene) is found in different immune cells, including macrophages, where it binds to PD-1 and marks it for ubiquitination, that leads to proteasomal degradation of the latter (40). This process reduces PD-1 expression on macrophages and aggravates their ability to phagocyte tumor cells, thereby inhibiting tumor growth (40). Phospholipase A2, membrane associated (PLA2G2A) is implicated in supporting an innate Th2-type immunity, which in turn boosts the early production of B-1 B cells (41).

A comprehensive 12-gene signature included *DRAM1*, *ELAPOR1*, *MAPT*, *TRIM58*, *UCHL1*, *CDKN2A*, *CYFIP2*, *AKT2*, *LINC00618*, *TTPA*, *TRIM46*, and *NOS2*, and belonged to Programmed Cell Death (PCD) pathways was used to estimate the response to immunotherapy in patients with EC. It was supposed that patients with increased PCD score may exhibit an attenuated response to immunotherapeutic modalities (42). The expression of *TRIM58*, which is a part of above-mentioned gene signature, positively correlated to the abundant accumulation of M2 macrophages and resting mast cells in the TME, and inversely correlated to the presence of Tfh in the TME in KRAS-mutant lung adenocarcinoma (43). An ubiquitin carboxy-terminal hydrolase L1 (*UCHL1*) expressed by macrophages facilitated the destruction of the phosphatidylinositol-4,5-bisphosphate 3-kinase, catalytic subunit alpha (PI3CA), via autophagic processes *in vivo*. This process resulted in the augmentation of the AKT pathway activity, which in turn, fostered the differentiation of macrophages towards the pro-inflammatory M1 phenotype (44). Cyclin-dependent kinase inhibitor 2A (*CDKN2A*) loss-of-function is associated with poorer clinical outcome in patients with advanced NSCLC treated with ICIs (45).

Interesting was the point that the long non-coding RNAs (lncRNAs) associated with cuproptosis, a process occurring by combining copper ions with the lipid-acylated components leading to cell death, can affect immune-related functions and the efficacy of immunotherapy (46). Recent study has demonstrated that molecular phenotypes related to cuproptosis, together with the scoring system known as CuproScore, can help in prognosis for patients with colorectal cancer and to predict the response to immunotherapy (47). These phenotypes included *FDX1*, *LIPT1*, *LIAS*, *DLD*, *DBT*, *GCSH*, *DLST*, *DLAT*, *PDHA1*, *PDHB*, *SLC31A1*, *ATP7A*, *ATP7B*, *CDKN2A*, *GLS*, and *MTF1* (47). Patients with gastric cancer categorized in the low cuproptosis-related prognosis signature subgroup exhibited elevated levels of tumor mutation burden (TMB), a higher incidence of MSI-H, and increased expression of PD-L1 in tumor (48). These factors were associated with a more favorable response to immunotherapy (48). Another study demonstrated that the subgroup of patients with low G Protein-Coupled Receptor (GPR) score (GPR-related genes) and high TME score had the highest proportion (31%) of patients responding to ICB therapy compared to those with high GPR and low TME (18). This subgroup also showed increased expression of several inhibitory immune markers, including *CTLA4*, *LAG3*, and *PDCD1*, as well as human leukocyte antigen (HLA) markers (18). Recent study indicated that the expression of *CCNE1* was significantly associated with TMB, MSI, the presence of neoantigens, and Immune Checkpoints in EC. This association could influence the responsiveness to immunotherapy treatment (49).

Thus, some essential genes play a fundamental role in maintaining the equilibrium in the TME, that is critical in the defining immunotherapy efficacy.

## 5 Proteomic signatures

One more prognostic model was constructed based on the protein expression of X1433EPSILON, Chk2-pT68, ER alpha, Fibronectin, PR, EPPK1, Annexin 1, Myosin IIA, and p16INK4a in EC tumor tissue (50). The expression of X1433EPSILON, ER-alpha, PR, Annexin 1, and Myosin IIA was associated with a better outcome, whereas the expression of Chk2-pT68, Fibronectin, EPPK1, and p16INK4a was associated with an unfavorable prognosis. The low-risk group exhibited higher proportions of CD8+ T cells, Tfh cells, and Tregs in the TME. Authors suggested that patients in the low-risk category were more likely to respond to immunotherapy compared to those in the high-risk group (50). Markers of immunotherapy response that are based on the immunohistochemical detection of particular molecules or cellular types were also revealed. In our recent study, we identified that in the TME of pMMR endometrial cancer, both the percentage of CD20+ B lymphocytes and the ratio of CD8-to-CD20 lymphocytes indicated responders to immune-targeted therapy and predicted favorable OS (51). In dMMR EC, non-responders generally had lower levels of CD8+ T cells, no terminally differentiated T cells, absent mature TLS and dendritic cells (52). A combination of four specific immune features (absence of PD-L1 and expression of TIM-3, IDO1, and LAG-3) accurately predicted ICI response (52).

## 6 Conclusions and future perspectives

Last years manifested emergency in patient stratification for the immunotherapy administration. For that, novel accurate predictive biomarkers must be identified. The data we collected in this review suggest that cells of the TME are critical in achieving high efficacy of immunotherapy in endometrial cancer. Thus, tumor infiltration with Tfh, cytotoxic T-cell, dysfunctional (CD8+PD-1+) or terminally dysfunctional (CD8+PD-1+TOX+) T cells, CXCL8^hi^IL1B^hi^ macrophages, as well as TLS presence, indicating “hot” tumor microenvironment, may be a strong indicator for beneficial immunotherapy response. Simultaneously, high TMB accompanied by mutations in critical genes increases the chance for a positive response to ICIs in patients with endometrial cancer. Systemic factors are also likely to make a significant contribution to the prognosis of disease progression. However, the literature lacks comprehensive studies on the predictive value of systemic factors in EC. Only the correlation of neutrophil-to-lymphocyte ratio (NLR) with the effectiveness of immunotherapies such as pembrolizumab, the combination of lenvatinib and pembrolizumab, nivolumab, and atezolizumab was studied. A pre-treatment NLR below 6 was associated with improved OS in recurrent EC patients received immunotherapy (53).

In recent years, using high-tech sequencing methods, various transcriptional signatures have been developed that reflect the functional activation of a number of cells in the TME. However, the role of gene signatures is controversial, since the use of sequencing methods involves the introduction of errors associated with the processing of big data, and, therefore, errors in the true predictive value of gene signatures.

We assure that to use described biomarker in clinics, the investigations on its predictive value should be started from the beginning of the clinical trial, followed by a stratified randomization process that comprehensively addresses the status of this biomarker. Moreover, to uncover applicable biomarker that able to predict immunotherapy effectiveness properly, precise molecular and cellular mechanisms underlying either tumor progression or tumor regression during treatment should be thoroughly investigated.

It should be noted that endometrial tumors, for which the administration of ICIs is applicable, accounts only 20-25% among all cases of EC. It is related to the nature of tumor and character of the TME. This fact indicates the exigence in the searching therapeutic strategies for proficient MMR tumors, as well as increasing the immunogenicity of tumor or the transition of “cold” into “hot” tumors. According to these demands, multiple clinical trials and pre-clinical studies demonstrated the success in using immunotherapy in combination with other types of therapies. These approaches include the combination immunotherapy, the use of immune checkpoint inhibitors with chemotherapy, with angiogenesis inhibitors, with radiotherapy, or epigenetic drugs. For example, pembrolizumab plus lenvatinib (a multitargeted tyrosine kinase inhibitor of vascular endothelial growth factor receptors 1 through 3, fibroblast growth factor receptors 1 through 4, platelet-derived growth factor receptor α, RET, and KIT) demonstrated longer PFS and OS than chemotherapy among patients with advanced EC that was not MSI-H/dMMR, followed by the approval of this combination by the FDA for this group of patients (54, 55).

Substantial reprogramming of the TME by several cytotoxic chemotherapeutic drugs and radiotherapy regiments was demonstrated to induce various immunostimulatory effects. Thus, chemotherapy may activate cytotoxic immune cells, enhance the presentation of tumor cell-specific antigens, and induce immunogenic cell death (56). Some preclinical models demonstrated convincingly that the efficacy of certain chemotherapeutic agents is higher in immunocompetent mice than in their immunodeficient counterparts (57). Moreover, immune stimulatory effects of chemotherapy can contribute to the transformation of “cold” to “hot” tumors in response to ICIs (58). All these evidences triggered clinical trials based on using combination chemotherapy and ICIs in EC patients (59, 60).

The mechanism of radiotherapy is associated with releasing tumor neoantigens and activation of adaptive immune response both locally in irradiated tumor site and in non-irradiated tumor metastases, known as abscopal effect, inducing immunogenic tumor cell death in the distant sites (61). Clinical trials with combination therapy based on ICIs and radiotherapy are limited for metastatic EC, but can be promising strategy to complementary control distant metastatic spread (61).

The leading-edge approach for treating EC is developing based on epigenetic modification inhibitors that are aimed to increase the immunogenicity of tumors and may help to facilitate the turning tumors from a “cold” into a “hot” ones (12, 58). The application of therapeutic epigenetic regulation combined with ICIs is under the clinical trials for several cancers, but not for endometrial cancer (10).

In summary, to improve the accuracy of the effectiveness of reported combinatorial approaches, it is urgently needed to unravel the intricate mechanisms of tumor immune microenvironment involvement in the pathogenesis of EC, and, more thoroughly, the mechanisms of the transition of “hot” to “cold” endometrial tumors. The inclusion of rigorous biomarker evaluation into clinical trials is required for the efficacious introduction of brand-new immunotherapeutic strategies and combinatory approaches targeting endometrial cancer. Hereby, the responsibility for the success of oncoming clinical trials goes outside the framework of simple evaluation of the efficacy of monotherapy or combination therapy; it encompasses an understanding of complex molecular ground that contributes to this success.
